# Motility Improvement of Biomimetic Trachea Scaffold via Hybrid 3D-Bioprinting Technology

**DOI:** 10.3390/polym13060971

**Published:** 2021-03-22

**Authors:** Young Soo Yu, Chi Bum Ahn, Kuk Hui Son, Jin Woo Lee

**Affiliations:** 1 Department of Health Sciences and Technology, GAIHST, Gachon University, Incheon 21999, Korea; koysm2@naver.com; 2 Department of Molecular Medicine, College of Medicine, Gachon University, Incheon 21999, Korea; cutemole@gmail.com; 3 Gil Medical Center, Department of Thoracic and Cardiovascular Surgery, Gachon University College of Medicine, Incheon 21565, Korea

**Keywords:** 3D printing, trachea scaffold, motility, electrospinning, adhesion

## Abstract

A trachea has a structure capable of responding to various movements such as rotation of the neck and relaxation/contraction of the conduit due to the mucous membrane and cartilage tissue. However, current reported tubular implanting structures are difficult to impelement as replacements for original trachea movements. Therefore, in this study, we developed a new trachea implant with similar anatomical structure and mechanical properties to native tissue using 3D printing technology and evaluated its performance. A 250 µm-thick layer composed of polycaprolactone (PCL) nanofibers was fabricated on a rotating beam using electrospinning technology, and a scaffold with C-shaped cartilage grooves that mimics the human airway structure was printed to enable reconstruction of cartilage outside the airway. A cartilage type scaffold had a highest rotational angle (254°) among them and it showed up to 2.8 times compared to human average neck rotation angle. The cartilage type showed a maximum elongation of 8 times higher than that of the bellows type and it showed the elongation of 3 times higher than that of cylinder type. In cartilage type scaffold, gelatin hydrogel printed on the outside of the scaffold was remain 22.2% under the condition where no hydrogel was left in other type scaffolds. In addition, after 2 days of breathing test, the amount of gelatin remaining inside the scaffold was more than twice that of other scaffolds. This novel trachea scaffold with hydrogel inside and outside of the structure was well-preserved under external flow and is expected to be advantageous for soft tissue reconstruction of the trachea.

## 1. Introduction

The trachea, a tubular organ that is a conduit that connects the larynx to the lungs, plays a role in the passage of sealed and exhaled air. The trachea can be damaged by stenosis, infections, trauma, congenital anomalies, and malignancies [1,2]. If a tracheal defect occurs, the mortality rate is very high; therefore, various studies have been conducted to address this problem. The treatment methods for the trachea are dependent on the size and length of the defect area. If the lesion is accompanied by a large tracheal stenosis and subglottic stenosis, methods such as tracheoplasty or tracheal autograft are performed. However, their operation requires complex procedure such as the tissue dividing, trimming, and suturing [3]. It is also accompanied by secondary damage during autologous tissue collection. Transplantation of donor tissues has been reported, but it is difficult to obtain adequate donor tissue. Even if a donor is secured and transplantation is successful, problems due to immune rejection can occur [4].

Recently, to address these obstacles, the development of bio-artificial organs based on tissue engineering technology has progressed [2,5,6,7]. A bio-artificial organ is an organ made by culturing cells on a three-dimensional bio-artificial scaffold and adding growth factors. In particular, 3D printing technology using biomaterials with excellent biocompatibility and organoids using organ-specific cell aggregations have drawn attention. Moreover, 3D bioprinting, one of 3D printing technologies, which uses bio-inks including cells, have recently attracted attention in organ reconstruction research [8,9,10,11]. In addition, to enhance the tissue reconstruction ability, surface treatments to improve cell adhesion and growth has been performed, and surface modifications using auxetic geometry have been studied to enhance an adhesion of cell and tissue [12,13]. Moreover, the extracellular matrix (ECM) has also been formed using hydration gel [14,15]. Further, 4D printing in which self-movement is added to 3D printing also has been studied [16,17,18].

To date, various studies using 3D printing have been conducted on trachea reconstruction including clinical trials [19,20,21,22,23], animal experiments [24,25,26,27,28,29,30,31,32,33,34,35,36,37,38], and basic experiments for measuring mechanical strength [39,40,41]. Since Zopf et al. (2013) first reported a case of a clinical trial using an airway external splint in an 8-week-old infant with bronchomalacia [19], Morrison et al. (2015) and Huang et al. (2016) used a 3D-printed polycaprolactone (PCL) external splint to treat bronchobronchiolasis [20,21]. Morrison et al. (2017) used an external splint made of polyetherketoneketone (PEKK) material and observed a therapeutic effect on tracheomalacia at the one-year follow-up [22]. Les et al. (2019) made an external splint using PCL/hydroxyapatite, and most patients showed improved breathing [23].

In animal studies, trachea defect grafts, circumferential tracheal segments and external splints were fabricated by 3D printing to investigate the possibility of trachea tissue reconstruction. In many cases, constructs were made of PCL, a biodegradable polymer, and their reconstruction performance was evaluated. In addition, we evaluated 3D-printed implants were made using poly(lactic acid) (PLA), poly(lactic-glycolic acid) (PLGA), PEKK, and polyurethane (PU), as well as the efficacy of trachea tissue reconstruction in various animals, including rabbits, sheep, and pigs [5,24,25,26,27,28,29,30,31,32,33,34,35,36,37,38].

As a basic study on the strength of tracheal constructs, Best et al. (2018) showed that 3D-printed tracheal ring implants can achieve desirable mechanical properties for humans [39]. In addition, Kang et al. (2019) and Pan et al. (2019) reported the strengths of polyurethane (PU) and PCL materials [40,41]. However, for the manufactured artificial organ/tissue to function in the human body, it must have a similar motility to the actual human organ/tissue. In particular, the trachea undergoes rotation and contraction/expansion movements, but the existing 3D printing technology imitates only the anatomical size, and studies on the biomechanics are insufficient [5,24,42,43]. For example, a cylinder type scaffold did not follow the bending and rotational movement of the neck, and the bellows type scaffold could follow the bending of the neck, but could not follow the rotational motion.

Here, to solve this problem, a trachea mimic scaffold with the appropriate anatomical, cell adhesion, and biomechanical properties through 3D printing and electrospinning of poly-ε caprolactone (PCL) was developed. The proposed ‘trachea type’ scaffold responded to the rotation and bending of the face and was designed to accommodate trachea lumen extension. In addition, a cartilage groove was added on the outer surface of the scaffold to provide an appropriate environment for the attachment and proliferation of chondrocytes. Furthermore, by adding an internal electrospun fiber, it provided an appropriate environment for attachment/proliferation of trachea mucosal cells. Actually, its motility and flexibility were verified through a rotation test, tensile strength test, compression test and 3 point bending test. A hydrogel attaching performance for chondrocytes and mucosal cells adhesion was evaluated using a circulation system and artificial respirator.

## 2. Materials and Methods

### 2.1. Materials

For 3D printing and electrospinning, biodegradable poly-ε caprolactone (M.W. 48,000–90,000; Sigma-Aldrich, Darmstadt, Germany) was purchased and used. Gelatin (pH 5.0–7.0; 60 kDa, Samchun Chemical, Seoul, Korea) was purchased and used as a hydrogel material to evaluate the adhesion to the inside and outside of the artificial trachea scaffold. To prepare a solution for electrospinning, chloroform (Sigma-Aldrich, Darmstadt, Germany) and methanol (Samchun Chemical, Seoul, Korea) were used as solvents for dissolving PCL.

### 2.2. FDM (Fused Deposition Modeling)-Based 3D Printing System

A fused deposition modeling (FDM) system (Geo Technology, Incheon, Korea) was used to fabricate an artificial trachea. The FDM printing method can realize a three-dimensional shape using the following procedure: a syringe is filled with a thermoplastic material, the melted material is printed through a syringe nozzle by applying heat and pressure, and 3D structures are manufactured by an accumulation of 2D shapes.

**Figure 1:**
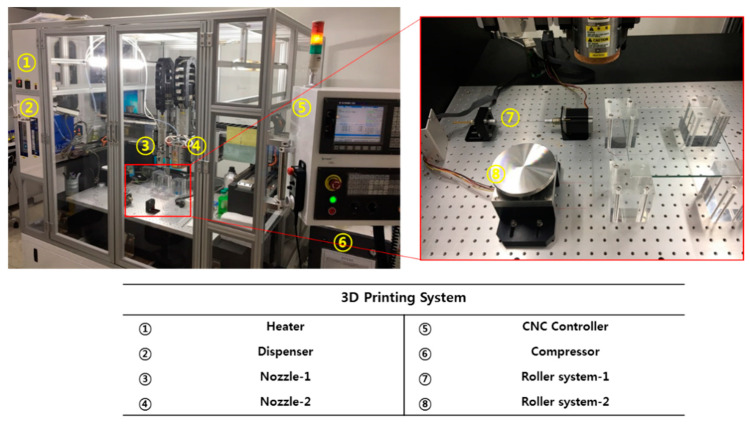
Configuration of the 3D printing system.

The 3D shape is first generated using computer-aided design (CAD) software and then code data needed to operate a 3D printing system are generated using computer-aided manufacturing (CAM) software. On the 3D printer, the nozzle moves along the X-, Y-, Z-, and W-axis following the operating path. Finally, the desired 3D shape is produced through the stacking of 2D shapes (Figure 1).

### 2.3. Electrospinning

**Figure 2:**
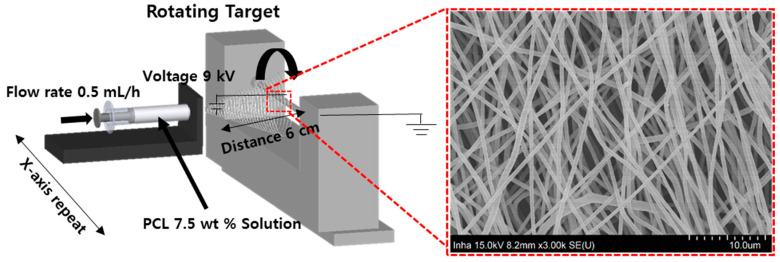
Schematic of electrospinning process.

Electrospun fibers were deposited on a rod using an electrospinning device (NanoNC, Seoul, Korea) (Figure 2). The flow rate of the electrospinning solvent was fixed at 0.5 mL/h, and the distance and angle between the spinning nozzle and the rod were 60 mm and 35°, respectively. The operating voltage was set to 9 kV, and the rotating speed of the rod was 10 rpm (revolutions per min). A mixture of chloroform and methanol (4:1) was used as the solvent to dissolve the PCL. The morphologies and sizes of the electrospun fibers were observed using an optical microscope (Optika, Bergamo, Italy).

### 2.4. Design and Manufacturing Process Using CAD (Computer Aided Design) and CAM (Computer Aided Manufacturing)

**Figure 3:**
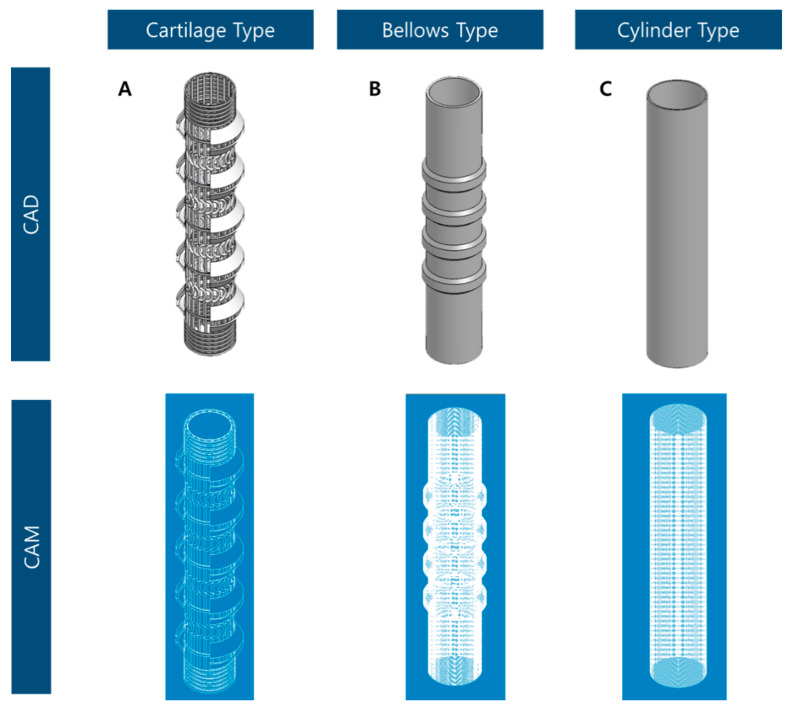
Design and 3D printing data for three types of trachea scaffolds: (**A**) cartilage type, (**B**) bellows type, and (**C**) cylinder type.

To develop an artificial trachea, CAD and CAM programs were used to design and manufacture the trachea. 3D shapes were designed by Solidworks (Dassault Systèmes, Vélizy-Villacoublay, France) and CAD software. To generate path data for 3D printing from the artificial trachea design, a Mastercam (CNC Software Inc., Tolland, CT, USA) which is CAM software, were used. After setting information such as nozzle size, printing speed, and printing temperature, the scaffolds were printed. In this study, three types of scaffold designs and code data for 3D printing were produced. The experimental group, which is named by ‘cartilage type’ was designed and coded (Figure 3A). The ‘bellows type’ and ‘cylinder type’ scaffolds (control groups) were designed and coded (Figure 3B,C).

### 2.5. Simulation Test

Cylinder type, bellows type and cartilage type scaffolds were designed using computer aided design (CAD) software (Solidworks 2017; Dassault Systemes, Vélizy-Villacoublay, France). The simulations were done using the finite element method tool of Solidworks. Properties of the PCL material necessary for the simulations were obtained from the manufacturer’s data sheet. To evaluate the structural behavior of the designed scaffolds, 3 point bending test was carried out, in which deformation was measured by applying a central load to the tubular scaffold with 5 mm inner diameter with both ends fixed. After applying a force from the top surface of the scaffold to half the diameter, the inner diameter of the lumen at the each scaffold was calculated.

### 2.6. Fabrication of Three Types of Trachea Scaffolds

**Figure 4:**
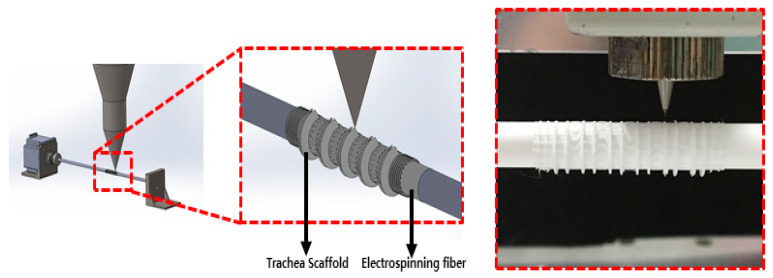
Schematic of the trachea scaffold fabrication.

The cartilage-type trachea scaffold was fabricated using a combination of an electrospun mat and a 3D-printed structure. First, at a flow rate of 0.5 mL/h, operating voltage of 9 kV, distance of 60 mm, and rotating speed of the rod of 10 rpm, we created an electrospun fibrous layer with 4 µm pores and 250 µm thickness. A PCL solution of 7.5% (*w*/*w*) was dissolved in a mixture of chloroform and methanol (4:1) as the electrospinning material. On the electrospun layer, we printed a cartilage-type scaffold with a pressure of 300 kPa, printing speed of 300 mm/min, and temperature of 363 K (90 °C). PCL was printed using a 25 G nozzle (inner diameter: 250 µm, MUSASHI Engineering, Tokyo, Japan) (Figure 4). The bellows-type and cylinder-type scaffolds were directly printed using a 3D printer by loading the code data prepared by CAD/CAM. The 3D printing conditions of the bellows-type and cylinder-type scaffolds were a pressure of 300 kPa, printing speed of 300 mm/min, and temperature of 363 K, similar to the printing condition of the cartilage-type scaffold.

### 2.7. Measurement of Rotation Angle

A torsion test was conducted based on the movement of the human neck using the developed trachea scaffold (cartilage type) and the existing bellows and cylinder-type trachea scaffolds. To measure the rotational angle, one side of the trachea was fixed using disk-type coupling (SRB-12C, Misumi, Seoul, Korea) with a diameter of 5 mm, and the other side, which was connected to the motor (A16k-G268, Autonics, Seoul, Korea) was rotated by 1 rpm (revolutions per min). The angle was measured until the inner diameter of the trachea was completely narrowed.

### 2.8. Measurements of Mechanical Properties

To evaluate the performance of the developed trachea scaffold, the tensile strength, compressive strength, Flexural stress, elastic modulus, and elongation ratio were measured using a universal testing machine (KDPI-130, KD Precision, Seoul, Korea). The testing load and speed were 500 m·kg·s^−2^ and 3.33 × 10^−5^ m/s, respectively. Three types of scaffolds, namely the cartilage, bellows, and cylinder type, had the same length of 30 mm and inner diameter of 5 mm. For tensile strength measurement, all specimens were fixed in the vertical direction using a universal testing machine. For compressive yield strength measurement, all specimens were set to the vertical direction. For the flexural test, a 30 mm long trachea scaffolds were placed between rigid supports and the center of the scaffold was pressed vertically from top to bottom by a sharp load cell. All experiments were repeated three times with different samples.

### 2.9. Hydrogel Residual on the Scaffolds in a Circulation Mock-Up System

To verify the adhesion of the hydrogel, a gelatin hydrogel (gelatin, 60 kDa, Samchun, Seoul, Korea) was printed on the outside of the trachea scaffolds. A gelatin hydrogel was prepared by adding 7.5 g gelatin and 50 mL of distilled water to a 50 mL conical tube (SPL Life Sciences, Korea) and mixing for 12 h at 300 K (27 °C) and 150 rpm. Then, the hydrogel was loaded into the 10 mL syringe of the 3D printer, and a curved nozzle with an inner diameter of 500 µm was mounted. Finally, the gelatin hydrogel was printed at 20 kPa pressure on the outside of the cylinder type, on the C shape ring position of the bellows type, and on the cartilage position of the cartilage-type trachea scaffold.

**Figure 5:**
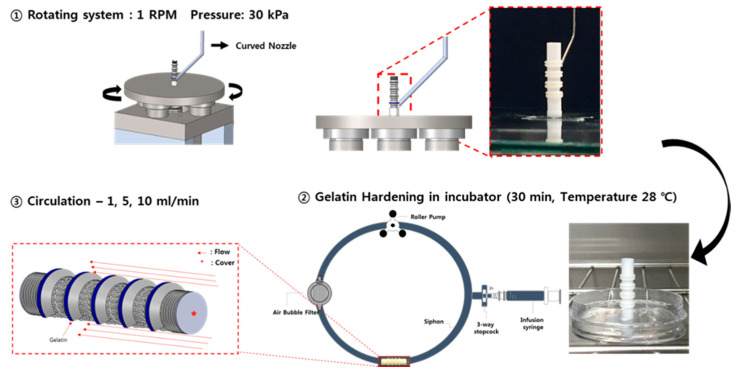
Experimental process using a circulation mock-up system.

As shown in Figure 5, the circulation mock-up system is composed of a closed circuit that connects each part with a tube, including an infusion syringe for fluid injection, a roller pump for flow generation, an air bubble filter for removing air from the tube, and a tracheal scaffold holder. 8 × 10^−5^ Kg of gelatin hydrogel was printed on the outer wall of three types of trachea structures using 3D bioprinting system. In the case of the bellows type, the hydrogel was printed on the groove of the outer surface, and in the case of the cylindrical structure, it was printed in the same position as the bellows type. In the case of the cartilage type, a hydration gel was printed in the pre-made groove where the cartilage would be located. An air bubble filter was added to keep the constant fluid flow rate by removing the bubbles present in the circulation system. The fluid circulation rate was set to 1, 5, and 10 mL/min by adjusting the rotational speed of the roller pump. After circulating distilled water for 30, 60, and 120 min, weights of residual hydrogel on the tracheal scaffolds were measured. A residual percentage of the hydrogel at each scaffold was calculated by a following Equation (1).

(1) $$\mathrm{Residual}\;\mathrm{of}\;\mathrm{the}\;\mathrm{hydrogel}\;\left(\%\right)=\frac{Scaffold\;weight\;\left(before\;circulation\right)-Scaffold\;weight\;\left(after\;circulation\right)}{Scaffold\;weight\;\left(before\;circulation\right)-Scaffold\;weight\;\left(before\;hydrogel\;printing\right)}\;\times \;100$$

### 2.10. Hydrogel Residual Inside of the Scaffold Assessed Using a Ventilation System

**Figure 6:**
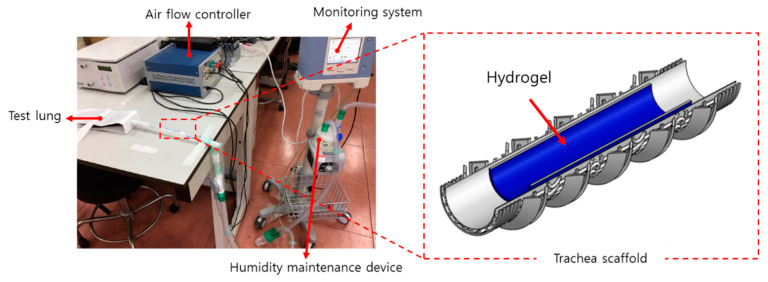
Configuration and experimental condition for the ventilation system.

To evaluate the effectiveness of the scaffold against internal tracheal stimuli such as breathing and coughing, hydrogel was printed inside the scaffolds, and the adhesion performance of the hydrogel was evaluated [44]. After generating the circulatory system shown in Figure 6, including clinical artificial respiration equipment (Trilogy100, Philips, Cambridge, MA, USA), the conditions of the system were set to a tidal volume of 170 mL, 30 breaths per minute, and an inspiration time of 1 s, similar to human breathing conditions. After the breathing tests were conducted for 1 and 2 days, the amount of residual hydrogel in the three types of scaffolds was observed.

### 2.11. Statistical Analysis

All the experiments were performed in triplicate, and the representative or average data are presented, unless otherwise stated. The data were analyzed using Prism (ver. 7; GraphPad Software, San Diego, CA, USA). The data within a given group or between groups were compared using a one-way analysis of variance (ANOVA). Significant differences are defined as * *p* < 0.05 or ** *p* < 0.001.

## 3. Results

### 3.1. Trachea Scaffold Fabrication

**Figure 7:**
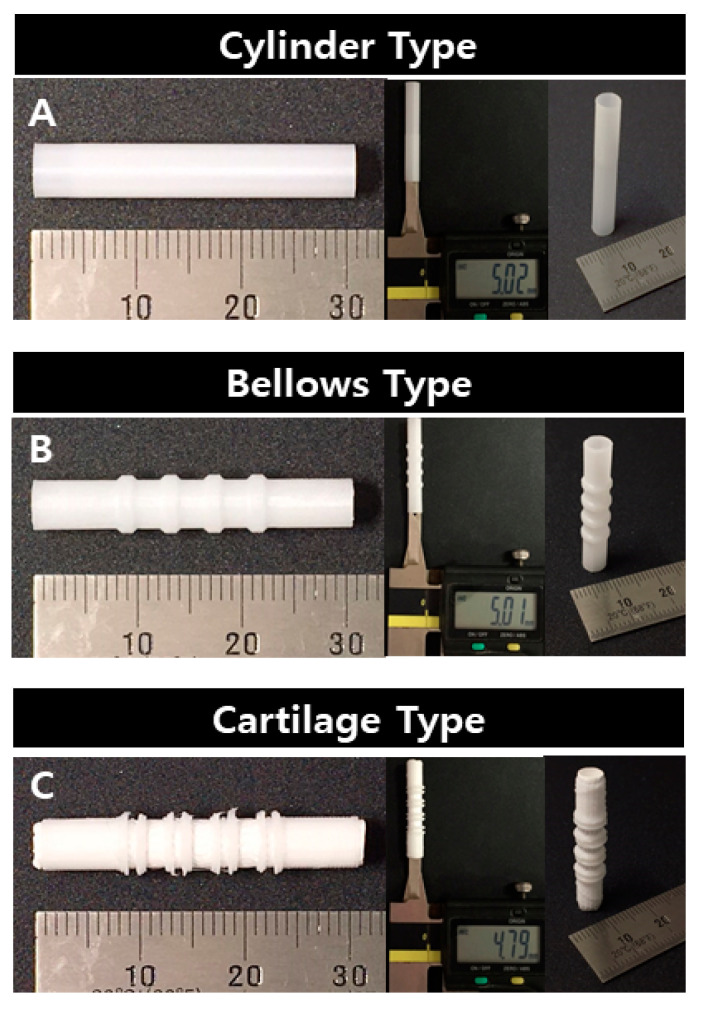
Fabrication results for trachea scaffolds: (**A**) cylinder type, (**B**) bellows type, (**C**) cartilage type.

For the scaffold length, all three designs were manufactured with the same length of 30 mm. For the scaffold diameter, there was a maximum difference of 4% between the design and manufacturing values. Because the cylinder type and the bellows-type scaffolds were made by 3D printing alone, they were manufactured with an error of less than 1% in the designed diameter of 5 mm (5.02 and 5.01 mm, respectively). However, in the case of the cartilage type, the inner diameter was 4.79 mm because electrospinning was first performed inside, and it was manufactured at 96% of the design value (Figure 7).

### 3.2. Simulation Result

**Figure 8:**
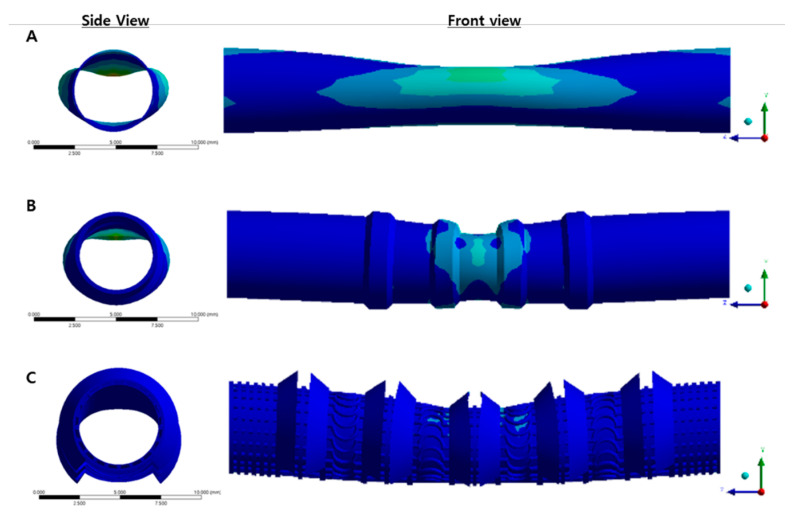
Simulation result for trachea scaffolds using 3 pointing bending test. (**A**) Cylinder type, (**B**) Bellows type, (**C**) Cartilage type.

In order to evaluate the structure behavior of the trachea scaffolds due to the external force and the breathing efficiency due to a lumen diameter change, three types of scaffolds with 5 mm inner diameter were modeled by Solidworks software, then a 3-point bending test was progressed (Figure 8). In case of a cartilage type that a tubular wall was stretchable by its wave pattern, the scaffold was bent with a minimal lumen deformation. As a result, even when bending occurred, the lumen diameter was maintained at 3.403 mm (68.06%). On the other hand, in the case of the cylinder type scaffold, which lacks an elasticity, a lumen diameter (2.798 mm, 55.96%) was reduced the most by external force. In the case of the bellows type, the value (3.005 mm, 60.10%) between the cartilage type and the cylinder type was shown.

### 3.3. Rotation Angles of Trachea Scaffolds

**Figure 9:**
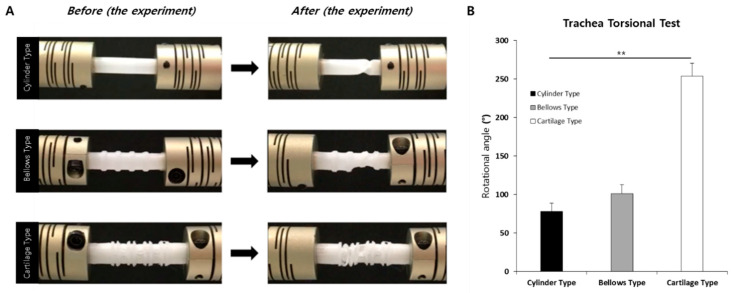
Torsional test results of the tracheas. (**A**) Experiment, (**B**) Rotation angle graph (** *p* < 0.001).

The human neck is rotated and bent as much as left and right rotation of 90°, 80° to 90° of flexion, and 70° of extension [45]. For the rotation test of the cylinder type scaffold, it was confirmed that the result was less than 13.3% of the human neck rotational angle, and it did not have suitable mobility as a trachea scaffold. For the bellows type, a rotation angle of approximately 101° was observed, which is similar to the rotation angle of the human neck (Figure 9). However, this is considered insufficient, considering that the rotational range is reduced as a result of fibrosis of the periphery during tissue reconstruction after transplantation surgery. On the other hand, it was confirmed that the developed cartilage type showed up to 2.8 times the rotation angle (254°) of the human average neck rotation angle.

### 3.4. Comparison of Mechanical Properties among Various Trachea Scaffolds

**Figure 10:**
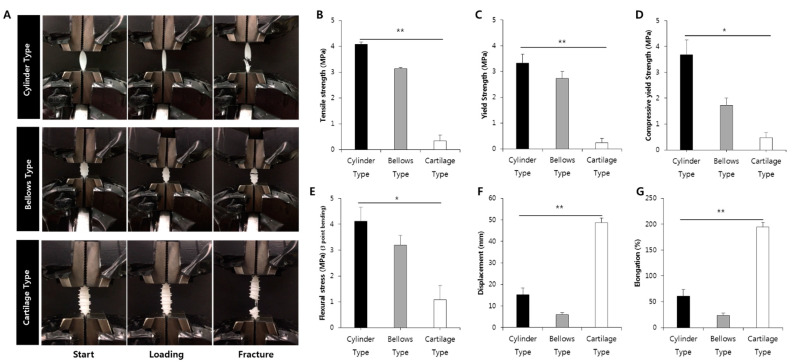
Results of mechanical properties measurements. (**A**) Tensile test process of each scaffold, (**B**) tensile strengths of three types of trachea scaffolds, (**C**) yield strengths of three types of trachea scaffolds, (**D**) compressive yield strengths of three types of trachea scaffolds, (**E**) flexural stress of three types of trachea scaffolds by 3 point bending test, (**F**) displacements of three types of trachea scaffolds, and (**G**) elongations of three types of trachea scaffolds. (* *p* < 0.05, ** *p* < 0.001).

To evaluate the kinetic performance of the trachea structure, the tensile strength, yield strength, compressive yield strength, flexural stress, displacement, and elongation of the scaffold were measured. It was confirmed that the cartilage type had low tensile strength and yield strength, compressive yield strength and flexural stress but the highest elongation and displacement values, which is advantageous for rotation and bending (Table 1). Specifically, cartilage type scaffold showed a maximum elongation of 8 times higher than that of the bellows type and it showed the elongation of 3 times higher than that of cylinder type. In the case of the cylinder and bellows types, because they were manufactured without pores through 3D printing, they possessed higher strength and stress than the cartilage-type scaffold (Figure 10).

### 3.5. Outside Flow Results for Hydrogel-Attached Scaffolds Using Circulation System

**Figure 11:**
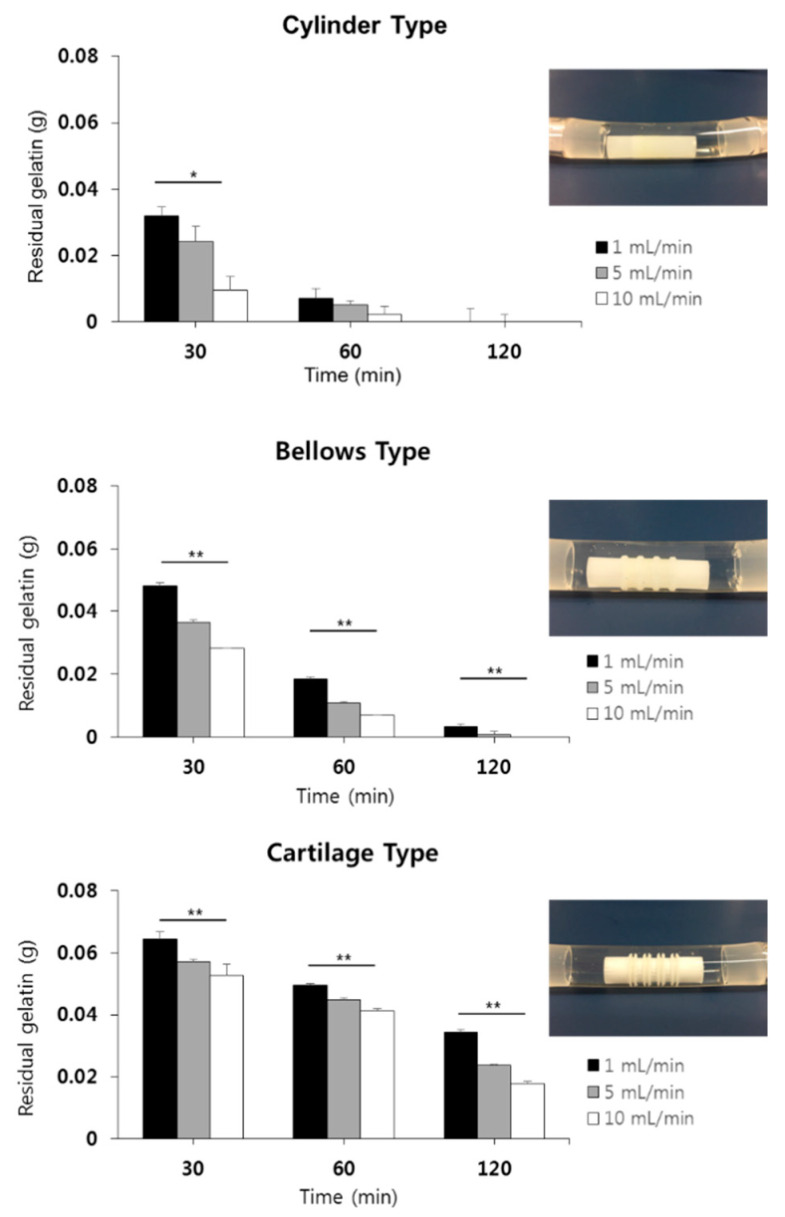
Measurement of residual hydrogel on trachea scaffolds using circulation system. (* *p* < 0.05, ** *p* < 0.001).

To evaluate the adhesion of the hydrogel to the outside of the scaffold, three types of trachea scaffolds with the hydrogel were inserted into a silicone tube with a diameter of 10 mm, and they were connected to the circulation equipment filled with distilled water. After 1, 5, and 10 mL/min of fluid flow was applied and circulated for 30, 60, and 120 min, the residual amount of the hydrogel was measured (Figure 11). When a flow rate of 1, 5, or 10 mL/min was applied, the hydrogel was completely dissolved within 120 min in the cylinder type, in which the flat outer surface was completely exposed to the fluid flow. For the bellows type, 4.0% (0.0032 g) of the gel remained after 120 min at 1 mL/min, and all of the gel was dissolved within 120 min at 5 and 10 mL/min, which was severe. On the other hand, for the cartilage type, which had an external shape that blocks the fluid flow and supports the hydrogel, 43.0% (0.0344 g), 29.6% (0.0237 g), and 22.2% (0.0178 g) hydrogel on average remained even after 120 min when flow rates of 1, 5, and 10 mL/min were applied, respectively. In the case of an actual tracheal transplant, the outside of the scaffold is in contact with the trachea muscle, which is not the same environment of rapid flow examined in this experiment. If a cell-laden hydrogel is mounted on the tracheal scaffold, the proposed cartilage-type scaffold will be advantageous.

### 3.6. Breathing Results for Hydrogel-Attached Scaffolds Using Clinical Ventilator System

**Figure 12:**
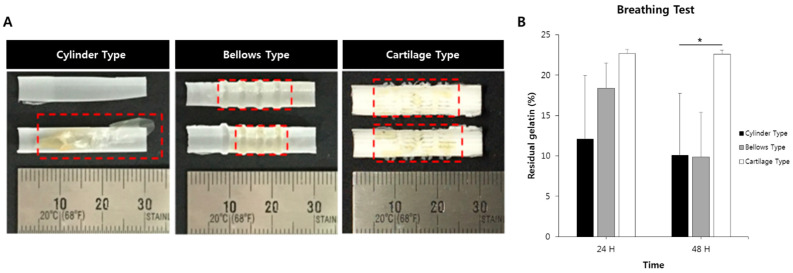
Measurement of residual hydrogel after the breathing test. (**A**) Observation of the luminal side after the breathing test and (**B**) gelatin residual after the breathing test. (* *p* < 0.05).

To evaluate adhesion ability on the inside of the artificial trachea, after printing the hydrogel on the lumens of tracheas, the same air volume generated in human breathing was applied through an artificial respirator, and the amount of hydrogel remaining inside the artificial trachea was measured. After preparing the 15% gelatin hydrogel, which was the same used in the circulation test, the hydrogel was printed onto lumens of three types of tracheas using a 3D printing system. The hydrogel-printed tracheas were inserted into a silicone tube with a 10 mm diameter (Sewoon Medical, Seoul, Korea) fitted to the length of the artificial trachea, and the tubes were connected to the artificial ventilation equipment. After the humidity control device installed in the artificial respirator was operated, air at 5.1 L/min was generated, and the residual amount of hydrogel was measured. As a result of the measurement, in the case of the cylinder type and the bellows type, the residual amount of hydrogel continuously decreased during the experimental period. However, the cartilage type maintained a meaningful amount of hydrogel during the experiment. On average, the gelatin hydrogel remained at 10.1% in the cylinder type and 9.9% in the bellows type and 22.6% in the cartilage type after 2 days of breathing testing (Figure 12). Based on the results of this experiment, the developed scaffold is expected to be an optimal structure for the generation of tracheal lumen mucosa using bio-ink.

## 4. Discussion

Although the trachea appears to be a simple tubular structure, it is a passage for oxygen and carbon dioxide exchange and is an important organ that discharges foreign substances and mucus generated during breathing. The lumen of the trachea, which consists of ciliated cells from the endothelium, is supported by the endothelial layer and outer cartilage in order to maintain the inner diameter of the airways during neck movements or swallowing. However, most trachea studies have not considered maintaining the tracheal lumen diameter, because most patch-type scaffolds are intended to treat partial tracheal defects.

Recently, Rehmani et al. (2017) implanted a 3D-printed PCL graft with biomimetic partial rings into pigs with a 4 cm anterior defect, and it was confirmed that 5 out of 7 transplanted animals survived 90 days after transplantation [30]. Goldstein et al. (2015) also used PLA as a scaffold seeded with rabbit chondrocytes for anterior tracheal defects in a live rabbit model with good mucosalization and without obstructive granulation tissue at three weeks [26]. Similarly, Jung et al. (2016) repaired anterior tracheal defects with 3D-printed polyurethane scaffolds and reported good results in patency with minimal stenosis [5].

Although various researchers have been trying to maintain the lumen diameter by developing a cylinder or bellows-type circumferential trachea scaffold, the results are still insufficient. A cylinder type has a one-dimensional hollow shape emulating the human trachea, but there is the disadvantage that it cannot achieve the movement of the native trachea. Because it is difficult for cells to grow in the inner layer with hydrophobicity, the cylinder-type graft is eventually blocked by stenosis. In a study by Xia et al. (2019), 3D-printed PCL circumferential tracheal segments were transplanted with autologous ear chondrocytes into goats, and all animals died after 100 days [38]. Compared to the cylinder type, the bellows-type scaffold is more effective as a tracheal structure than a cylindrical scaffold because the internal diameter of the trachea is not narrowed when the circumferential tracheal segment is bent, so the flow of air during breathing is not disturbed. As a result, Park et al. (2018) achieved complete re-epithelialization of the entire luminal surface within 2 months in a rabbit model [33]. However, like a cylindrical scaffold, it is not a structure that can withstand torsion, i.e., the rotation of the neck. However, the cartilage type that was developed in this study could compensate for the limitations of the previous trachea scaffolds by considering the anatomical shape, and it was developed as a shape that can fix the hydrogel for cartilage tissue reconstruction in an appropriate position.

To evaluate a motility performance of our trachea scaffold, comparison between the existing tracheal scaffolds (bellows type and cylinder type) and the developed trachea scaffold (cartilage type) were performed. In the rotation angle test, the developed cartilage type scaffold (254°) showed more than 2.8 times higher than the rotation angle of the human neck. On the other hand, the bellows type showed a rotation angle of 101°, and the cylinder type was 78°. Although the bellows type was slightly better than the average human neck rotation angle, this is considered an insufficient value considering the decrease in rotation performance due to the fibrous tissue that may occur around the scaffold during the treatment. In the case of the cylinder type, although it serves as a passage for breathing air, which is one of the purposes of the trachea, its rotation angle could not follow the rotation of the neck. Therefore, it was confirmed that our scaffold, with an excellent rotation angle, was effective for the recovery of the true function of the trachea.

The tensile testing results confirmed that the developed cartilage type scaffold had a maximum displacement and elongation eight times higher than that of the cylinder and bellows types. Although the tensile strength and compressive strength of our cartilage scaffold were lower than that of the cylinder and bellows trachea scaffolds, there was no problem with the mechanical strength, because no damage to the structure was found during the circulation and breath ventilation tests. In fact, in trachea, since the cartilage is mainly responsible for mechanical properties, and the implanted PCL scaffold will be degraded and disappeared, it seems the right direction to try to regenerate the cartilage in a short time to secure mechanical properties. In addition, a simulation result in which the inner diameter of our trachea was reduced to the smallest by external forces showed that our design was advantageous for supplying sufficient oxygen for breathing. Therefore, in the manufacturing of an artificial trachea scaffold, a highly flexible structure that exhibits strength in rotation and bending is considered advantageous for trachea reconstruction.

Since the trachea cartilage plays a supporting role in the human trachea, the generation of cartilage tissue is important to maintain the strength of the implanted trachea structure. Therefore, the printed cartilage bio-ink needs to be well-preserved in its original position during trachea reconstruction using bioprinting technology. For this purpose, in this study, a trachea scaffold was successfully fabricated, including the structure to prevent detachment of the hydrogel, which is a base material of bio-ink, inside and outside of the scaffold. To evaluate the adhesion performance of the hydrogel, a circulation mockup system and an artificial respirator-based experimental system were established, and hydrogel detachment experiments were performed on three types of trachea scaffolds. When we measured the hydrogel adhesion performance on the outside of the scaffolds with bellow-type, cylinder-type, and cartilage-type scaffolds, the cartilage-type trachea showed the best result, with 34% more hydrogel attached than the bellows type. Because the cylinder type scaffold has a smooth outer surface, all hydrogels attached on the outer surface were separated and washed within 2 h, even at a low flow. Meanwhile, various methods, such as intra-tracheal printing using bio-ink, direct culture using cell suspension, and cell migration from the original organ have been performed to reconstruct the internal mucosa layer. At those methods, most important factor is to enhance the ability of cells to adhere inside the trachea. In this study, this issue was solved by generating a cell adhesion layer using electrospun nanofibers inside the trachea scaffold. At the detaching test of the hydrogel attached on the scaffold lumen using artificial respirator-based experimental system, the developed trachea scaffold showed a two times higher hydrogel adhesion than the existing scaffolds for a long experimental period (48 h). From detaching results of the hydrogel attached to the outside and inside of the scaffolds, it was confirmed that the superiority of the developed scaffold.

## 5. Conclusions

In the development of a trachea scaffold, dynamic performance evaluation that considers neck rotation and adhesion wherein actual cells are attached inside and outside of the scaffold is a novel approach. Therefore, in this study, a trachea mimic scaffold with the appropriate anatomical, cell adhesion, and biomechanical properties through 3D printing and electrospinning of PCL was developed. The new trachea scaffold with high rotation angle, elongation, displacement and low deformation in an inner diameter will improve the structural stability and flow rate of air. A high hydrogel attachment performance at the inside and outside of the scaffold will help trachea reconstruction by increasing cell preservation. If in vitro cell adhesion and proliferation performances of our bioprinted scaffold and in vivo study are progressed in the near future, it will be a new possibility for a trachea stenosis treatment.

## Figures and Tables

**Table 1:** Mechanical properties of various types of trachea scaffolds.

Mechanical Property	Type of Trachea Scaffold		
	Cylinder Type	Bellows Type	Cartilage Type
Tensile strength (kPa)	4087 ± 76	3143 ± 40	470 ± 140
Yield strength (kPa)	3320 ± 350	2740 ± 270	237 ± 166
Compressive yield strength (kPa)	3672 ± 586	1731 ± 282	475 ± 193
Flexural Stress (kPa)	4117 ± 550	3191 ± 384	1079 ± 556
Elongation ratio (%)	60.83 ± 12.62	23.77 ± 3.94	194.71 ± 9.13
Displacement (mm)	15.20 ± 3.15	5.94 ± 0.99	48.68 ± 2.28

## Data Availability

The data presented in this study are available on request from the corresponding author.

## References

[1] Griffith L.G., Naughton G. (2002). Tissue engineering e current challenges and expanding opportunities. Science.

[2] Ghezzi C.E., Marelli B., Donelli I., Alessandrino A., Freddi G., Nazhat S.N. (2014). The role of physiological mechanical cues on mesenchymal stem cell differentiation in an airway tract-like dense collagen–silk fibroin construct. Biomaterials.

[3] Lee J.-C., Kim M.-S., Kim D.-J., Park D.-H., Lee I.-W., Roh H.-J., Lee B.-J., Kim Y.-A., Ko S., Sung E.-S. (2019). Subglottic stenosis in children: Our experience at a pediatric tertiary center for 8 years in South Korea. Int. J. Pediatr. Otorhinolaryngol..

[4] Ochando J., Charron D., Baptista P.M., Uygun B.E. (2017). Immune responses to bioengineered organs. Curr. Opin. Organ Transp..

[5] Jung S.Y., Lee S.J., Kim H.Y., Park H.S., Wang Z., Kim H.J., Yoo J.J., Chung S.M., Kim H.S. (2016). 3D printed polyurethane prosthesis for partial tracheal reconstruction: A pilot animal study. Biofabrication.

[6] Fishman J.M., Wiles K., Lowdell M.W., De Coppi P., Elliott M.J., Atala A., Birchall M.A. (2014). Airway tissue engineering: An update. Expert Opin. Biol. Ther..

[7] Maughan E.F., Butler C.R., Crowley C., Teoh G.Z., Den Hondt M., Hamilton N.J., Hynds R.E., Lange P., Ansari T., Urbani L. (2017). A comparison of tracheal scaffold strategies for pediatric transplantation in a rabbit model. Laryngoscope.

[8] Bernhard J.-C., Isotani S., Matsugasumi T., Duddalwar V., Hung A.J., Suer E., Baco E., Satkunasivam R., Djaladat H., Metcalfe C. (2016). Personalized 3D printed model of kidney and tumor anatomy: A useful tool for patient education. World J. Urol..

[9] Lee J.W., Choi Y.-J., Yong W.-J., Pati F., Shim J.-H., Kang K.S., Kang I.-H., Park J., Cho D.-W. (2016). Development of a 3D cell printed construct considering angiogenesis for liver tissue engineering. Biofabrication.

[10] Vukicevic M., Mosadegh B., Min J.K., Little S.H. (2017). Cardiac 3D printing and its future directions. JACC Cardiovasc. Imaging.

[11] Askari M., Naniz M.A., Kouhi M., Saberi A., Zolfaghariane A., Bodaghi M. (2021). Recent progress in extrusion 3D bioprinting of hydrogel biomaterials for tissue regeneration: A comprehensive review with focus on advanced fabrication techniques. Biomater. Sci..

[12] Ghavidelnia N., Bodaghi M., Hedayat R. (2021). Femur auxetic meta-implants with tuned micromotion distribution. Materials.

[13] Lee J.W., Soman P., Park J.H., Chen S., Cho D.-W. (2016). A tubular biomaterial construct exhibiting a negative Poisson’s ratio. PLoS ONE.

[14] Ahn G., Min K.-H., Kim C., Lee J.-S., Kang D., Won J.-Y., Cho D.-W., Kim J.-Y., Jin S., Yun W.-S. (2017). Precise stacking of decellularized extracellular matrix based 3D cell-laden constructs by a 3D cell printing system equipped with heating modules. Sci. Rep..

[15] Jang J., Park H.-J., Kim S.-W., Kim H., Park J.Y., Na S.J., Kim H.J., Park M.N., Choi S.H., Park S.H. (2017). 3D printed complex tissue construct using stem cell-laden decellularized extracellular matrix bioinks for cardiac repair. Biomaterials.

[16] Zolfagharian A., Kaynak A., Bodaghi M., Kouzani A.Z., Gharaie S., Nahavandi S. (2020). Control-based 4D printing: Adaptive 4D-printed systems. Appl. Sci..

[17] Bodaghi M., Damanpack A.R., Liao W.H. (2016). Self-expanding/shrinking structures by 4D printing. Smart Mater. Struct..

[18] Kim S.H., Seo Y.B., Yeon Y.K., Lee Y.J., Park H.S., Sultan M.T., Lee J.M., Lee J.S., Lee O.J., Hong H. (2020). 4D-bioprinted silk hydrogels for tissue engineering. Biomaterials.

[19] Zopf D.A., Hollister S.J., Nelson M.E., Ohye R.G., Green G.E. (2013). Bioresorbable airway splint created with a three-dimensional printer. N. Engl. J. Med..

[20] Morrison R.J., Hollister S.J., Niedner M.F., Mahani M.G., Park A.H., Mehta D.K., Ohye R.G., Green G.E. (2015). Mitigation of tracheobronchomalacia with 3D-printed personalized medical devices in pediatric patients. Sci. Transl. Med..

[21] Huang L., Wang L., He J., Zhao J., Zhong D., Yang G., Guo T., Yan X., Zhang L., Li D. (2016). Tracheal suspension by using 3-dimensional printed personalized scaffold in a patient with tracheomalacia. J. Thorac. Dis..

[22] Morrison R.J., Sengupta S., Flanangan C.L., Ohye R.G., Hollister S.J., Green G.E. (2017). Treatment of severe acquired tracheomalacia with a patient-specific, 3D-printed, permanent tracheal splint. JAMA Otolaryngol..

[23] Les A.S., Ohye R.G., Filbrun A.G., Ghadimi Mahani M., Flanagan C.L., Daniels R.C., Kidwell K.M., Zopf D.A., Hollister S.C., Green G.E. (2019). 3D-printed, externally-implanted, bioresorbable airway splints for severe tracheobronchomalacia. Laryngoscope.

[24] Zopf D.A., Flanagan C.L., Wheeler M., Hollister S.J., Green G.E. (2014). Treatment of severe porcine tracheomalacia with a 3-dimensionally printed, bioresorbable, external airway splint. JAMA Otolaryngol..

[25] Park J.H., Park J.Y., Nam I.-C., Hwang S.-H., Kim C.-S., Jung J.W., Jang J., Lee H., Choi Y., Park S.H. (2015). Human turbinate mesenchymal stromal cell sheets with bellows graft for rapid tracheal epithelial regeneration. Acta Biomater..

[26] Goldstein T.A., Smith B.D., Zeltsman D., Grande D., Smith L.P. (2015). Introducing a 3-dimensionally printed, tissue-engineered graft for airway reconstruction: A pilot study. JAMA Otolaryngol..

[27] Kaye R., Goldstein T., Aronowitz D., Grande D.A., Zeltsman D., Smith L.P. (2017). Ex vivo tracheomalacia model with 3D-printed external tracheal splint. Laryngoscope.

[28] Lee D.Y., Park S.A., Lee S.J., Kim T.H., Oh S.H., Lee J.H., Kwon S.K. (2016). Segmental tracheal reconstruction by 3 D-printed scaffold: Pivotal role of asymmetrically porous membrane. Laryngoscope.

[29] Bhora F.Y., Lewis E.E., Rehmani S.S., Ayub A., Raad W., Al-Ayoubi A.M., Lebovics R.S. (2017). Circumferential three-dimensional–printed tracheal grafts: Research model feasibility and early results. Ann. Thorac. Surg..

[30] Rehmani S.S., Al-Ayoubi A.M., Ayub A., Barsky M., Lewis E., Flores R., Lebovics R., Bhora F.Y. (2017). Three-dimensional-printed bioengineered tracheal grafts: Preclinical results and potential for human use. Ann. Thorac. Surg..

[31] Gao M., Zhang H., Dong W., Bai J., Gao B., Xia D., Feng B., Chen M., He X., Yin M. (2017). Tissue-engineered trachea from a 3D-printed scaffold enhances whole-segment tracheal repair. Sci. Rep..

[32] Taniguchi D., Matsumoto K., Tsuchiya T., Machino R., Takeoka Y., Elgalad A., Gunge K., Takagi K., Taura Y., Hatachi G. (2018). Scaffold-free trachea regeneration by tissue engineering with bio-3D printing. Interact. Cardiovasc. Thorac. Surg..

[33] Park J.H., Park J.Y., Nam I.-C., Ahn M., Lee J.Y., Choi S.H., Kim S.W., Cho D.-W. (2018). A rational tissue engineering strategy based on three-dimensional (3D) printing for extensive circumferential tracheal reconstruction. Biomaterials.

[34] Park J.-H., Yoon J.-K., Lee J.B., Shin Y.M., Lee K.-W., Bae S.-W., Lee J., Yu J., Jung C.-R., Youn Y.-N. (2019). Experimental tracheal replacement using 3-dimensional bioprinted artificial trachea with autologous epithelial cells and chondrocytes. Sci. Rep..

[35] Gao B., Jing H., Gao M., Wang S., Fu W., Zhang X., He X., Zheng J. (2019). Long-segmental tracheal reconstruction in rabbits with pedicled Tissue-engineered trachea based on a 3D-printed scaffold. Acta Biomater..

[36] Kaye R., Goldstein T., Grande D.A., Zeltsman D., Smith L.P. (2019). A 3-dimensional bioprinted tracheal segment implant pilot study: Rabbit tracheal resection with graft implantation. Int. J. Pediatr. Otorhinolaryngol..

[37] Machino R., Matsumoto K., Taniguchi D., Tsuchiya T., Takeoka Y., Taura Y., Moriyama M., Tetsuo T., Oyama S., Takagi K. (2019). Replacement of rat tracheas by layered, trachea-like, scaffold-free structures of human cells using a bio-3D printing system. Adv. Healthc. Mater..

[38] Xia D., Jin D., Wang Q., Gao M., Zhang J., Zhang H., Bai J., Feng B., Chen M., Huang Y. (2019). Tissue-engineered trachea from a 3D-printed scaffold enhances whole-segment tracheal repair in a goat model. J. Tissue Eng. Regen. Med..

[39] Best C.A., Pepper V.K., Ohst D., Bodnyk K., Heuer E., Onwuka E.A., King N., Strouse R., Grischkan J., Breuer C.K. (2018). Designing a tissue-engineered tracheal scaffold for preclinical evaluation. Int. J. Pediatr. Otorhinolaryngol..

[40] Kang Y., Wang C., Qiao Y., Gu J., Zhang H., Peijs T., Kong J., Zhang G., Shi X. (2019). Tissue-engineered trachea consisting of electrospun patterned sc-PLA/GO-g-IL fibrous membranes with antibacterial property and 3D-printed skeletons with elasticity. Biomacromolecules.

[41] Pan S., Zhong Y., Shan Y., Liu X., Xiao Y., Shi H. (2019). Selection of the optimum 3D-printed pore and the surface modification techniques for tissue engineering tracheal scaffold in vivo reconstruction. J. Biomed. Mater. Res. A.

[42] Park J.H., Hong J.M., Ju Y.M., Jung J.W., Kang H.-W., Lee S.J., Yoo J.J., Kim S.W., Kim S.H., Cho D.-W. (2015). A novel tissue-engineered trachea with a mechanical behavior similar to native trachea. Biomaterials.

[43] Park J.H., Jung J.W., Kang H.-W., Joo Y.H., Lee J.-S., Cho D.-W. (2012). Development of a 3D bellows tracheal graft: Mechanical behavior analysis, fabrication and an in vivo feasibility study. Biofabrication.

[44] Minnich D.J., Mathisen D.J. (2007). Anatomy of the trachea, carina, and bronchi. Thorac. Surg. Clin..

[45] Swartz E.E., Floyd R., Cendoma M. (2005). Cervical spine functional anatomy and the biomechanics of injury due to compressive loading. J. Athl. Train..

